# Overexpression of SLC44A4 suppresses ferroptosis and reduces lipid peroxidation *via* noncanonical NF-κB signaling in a NIK-dependent manner

**DOI:** 10.1080/07853890.2026.2677931

**Published:** 2026-05-25

**Authors:** Tian Li, Yan Xu, Wenyu Song, Huafan Chang, Junqi He, Suli Liu, Zhicheng Liu, Yuance Xu, Qin Yao, Kejuan Song

**Affiliations:** Department of Obstetrics and Gynecology, The Affiliated Hospital of Qingdao University, Qingdao, China

**Keywords:** Ovarian cancer, lipid peroxidation, ferroptosis, NF-κB signaling

## Abstract

**Background:**

Ovarian cancer (OC) is one of the most common gynecologic malignancies worldwide and is characterized by a high recurrence rate driven by drug resistance, contributing to poor prognosis and increased mortality. Consequently, there is an urgent need to develop novel therapeutic strategies to improve patient outcomes. Although SLC44A4 is known to be highly expressed in various tumor types, its precise functional role and underlying mechanisms in OC remain largely unexplored.

**Objective:**

This study aims to investigate the effects and underlying mechanisms of SLC44A4 overexpression on the biological behavior of OC cells.

**Results:**

SLC44A4 enhances the proliferation and migration of OC cells both *in vitro* and *in vivo*. Moreover, SLC44A4 overexpression reduces lipid peroxidation, suppresses ferroptosis, and is associated with activation of noncanonical NF-κB signaling, resulting in decreased sensitivity to the ferroptosis inducer erastin.

**Conclusion:**

Our findings suggest that SLC44A4 overexpression reduces lipid peroxidation and suppresses ferroptosis, with these effects being linked to the activation of noncanonical NF-κB signaling. Thus, SLC44A4 may serve as a potential target for modulating ferroptosis in OC; however, further validation, including loss-of-function studies and patient-derived sample analyses, is required.

## Introduction

Ovarian cancer (OC) is a leading gynecological malignancy typically diagnosed in postmenopausal women. Recent epidemiological data, however, indicate a concerning trend toward earlier onset, with increasing cases identified in younger women [1]. Due to the deep pelvic location of the ovaries, early-stage OC often remains asymptomatic. Consequently, most patients present with pelvic masses, abdominal pain, or abnormal vaginal bleeding, and diagnosis frequently occurs at advanced stages. Although cytoreductive surgery combined with platinum-based chemotherapy and immunotherapy significantly improves 5-year survival, approximately 80% of patients experience disease recurrence due to platinum resistance. This emphasizes the urgent need for new therapeutic strategies targeting refractory OC [2,3].

Tumor cells undergo substantial metabolic alterations, including dysregulated glycolysis [4], abnormal lipid metabolism [5], and disrupted amino acid homeostasis [6], to sustain proliferation and survival. Among these alterations, reprogrammed lipid metabolism has emerged as a hallmark of cancer-associated metabolic changes. Accumulating evidence indicates that lipid metabolism pathways, such as fatty acid oxidation and lipogenesis, are closely linked to ferroptosis. Targeting these metabolic vulnerabilities may thus provide novel therapeutic options for chemoresistant malignancies [7].

Ferroptosis, a recently identified form of regulated cell death, is characterized by iron-dependent lipid peroxidation resulting from excessive reactive oxygen species (ROS) accumulation. This reflects a critical imbalance between oxidative stress and antioxidant defense mechanisms [8,9]. Mechanistically distinct from other cell death pathways, ferroptosis strongly associates with suppression of antioxidant defenses [10].

The NF-κB signaling pathway, involving key transcription factors, centrally regulates inflammatory responses, cell proliferation, differentiation, and survival. Emerging evidence also suggests that NF-κB signaling might influence ferroptosis through metabolic reprogramming [11,12].

SLC44A4 is located on chromosome 6 and belongs to the choline transporter-like (CTL) family. Although SLC44A1 and SLC44A2 have demonstrated choline transport activity, the precise biological functions of SLC44A4 remain unclear [13,14]. Current evidence indicates possible involvement in acetylcholine synthesis and transport, as well as uptake of thiamine pyrophosphate (the active form of vitamin B1) [15,16]. SLC44A4 displays tissue-specific expression patterns in various malignancies. It is positively expressed in over 90% of pancreatic and prostate carcinomas, with expression levels positively correlated with tumor grade [17]. In colorectal adenocarcinoma, it is downregulated, suggesting a potential role in regulating the tumor microenvironment [18]. It is upregulated in leptomeningeal metastases of small-cell lung cancer, potentially linked to chemoresistance [19]. In clear cell renal cell carcinoma, it serves as an NK cell-related gene with reduced expression, providing significant prognostic value [20]. Despite its established roles in multiple cancers, the biological significance of SLC44A4 in OC remains unexplored. Given the unique metabolic features of OC and its susceptibility to ferroptosis, investigating SLC44A4 in ovarian carcinogenesis holds substantial theoretical and clinical importance.

In this study, we found that SLC44A4 overexpression correlated with reduced lipid peroxidation and ferroptosis in OC cells, associated with activation of non-canonical NF-κB signaling. Analysis of public databases (TCGA) revealed that elevated SLC44A4 expression correlated with advanced disease stage and poorer patient survival. Therefore, SLC44A4 warrants further exploration as a potential prognostic marker in epithelial OC.

## Materials and methods

### Cell culture and lentiviral transfection

Human epithelial ovarian cancer (EOC) cell lines OVCAR3 (RRID: CVCL_0465) and A2780 (RRID: CVCL_0134), along with the normal ovarian epithelial cell line IOSE80 (RRID: CVCL_5546), were obtained from the Chinese Academy of Sciences (Shanghai, China). All cell lines were authenticated by STR profiling within the past three years [21] and tested negative for mycoplasma contamination [22]. IOSE80 cells were cultured in RPMI 1640, whereas EOC cells were cultured in DMEM; both media were supplemented with 10% fetal bovine serum (FBS). Cells were maintained at 37 °C in a humidified atmosphere containing 5% CO_2_. Lentiviral particles encoding SLC44A4 (GV492 vector; Ubiquitin promoter for SLC44A4; CBh promoter for IRES-puromycin; C-terminal 3× FLAG tag) were purchased from GeneChem (Shanghai, China). OVCAR3 and A2780 cells were transduced at an MOI of 30, followed by selection with 2.5 μg/mL puromycin for 7 days to generate stable pooled populations.

### Quantitative real-time PCR (RT-qPCR)

Total RNA was extracted using TRIzol reagent (Takara). RNA concentration and purity were measured using a NanoDrop 2000 spectrophotometer. One microgram of RNA was reverse-transcribed into cDNA using the PrimeScript RT Reagent Kit (Takara) with random hexamers in a 20 μL reaction. RT-qPCR was performed using TB Green Premix Ex Taq II (Takara) on a CFX96 Real-Time PCR System (Bio-Rad). Primer sequences used were:

SLC44A4 forward: 5′‑GCGGCAGCGGATTCGTATTG‑3′, reverse: 5′‑ATGAGGAGGAGGACAAAGGTGAC‑3′
β‑actin (internal control) forward: 5′‑CATGTACGTTGCTATCCAGGC‑3′, reverse: 5′‑CTCCTTAATGTCACGCACGAT‑3′

Thermal cycling consisted of initial denaturation at 95 °C for 1  min, followed by 40 cycles at 95 °C for 20 s and 60 °C for 1  min. Melting curve analysis ranged from 65 °C to 95 °C (increments of 0.5 °C, 5 s per step). Amplification efficiency (90%–110%) was confirmed using standard curves generated from five-step, tenfold serial dilutions. No-template controls (NTC) and no-reverse transcription controls (no-RT) yielded Ct values greater than 35. Three biological replicates (independent cell passages), each with three technical replicates, were analyzed. Relative gene expression was calculated by the 2^−ΔΔCt^ method, normalized to β-actin. All qPCR experiments complied with MIQE guidelines [23].

### Western blotting

Cells were lysed using SDS lysis buffer supplemented with protease and phosphatase inhibitors. Protein samples (20 μg) were separated by 10% or 12.5% SDS-PAGE and transferred onto PVDF membranes (Millipore). Membranes were blocked in 5% non-fat milk in TBST for 2  h at room temperature and incubated overnight at 4 °C with the following primary antibodies (dilutions indicated):

HUABIO: SLC44A4 (RRID:AB_3071635, 1:500), CPT1A (clone PSH04-00, HA750923, 1:1000), ACC1 (RRID:AB_3072827, 1:1000), ACSL4 (RRID:AB_3071100, 1:1000), NOX1 (ER1913-99, 1:1000).

ZENBIO: FADS1 (822332, 1:1000), SCD1 (clone R04-2K1, R25675, 1:1000), FASN (clone 2F9-B2-B6, 200194, 1:1000), FTH1 (381204, 1:1000), SLC7A11 (R382036, 1:1000).

Proteintech: LPCAT3 (RRID:AB_2918639, 1:1000), GPX4 (RRID:AB_2909469, 1:1000), TFRC (clone 242122B2, 84766-4-RR, 1:1000).

UpingBio: IKKα (YP-mAb-14780, 1:1000), p52/p100 (YP-mAb-00788, 1:1000), TRAF3 (YP-mAb-04251, 1:1000).

Servicebio: GAPDH (GB12002, 1:5000).

After washing, membranes were incubated for 2 h at room temperature with HRP-conjugated goat anti-rabbit or anti-mouse IgG (1:5000, Proteintech). Bands were visualized using enhanced chemiluminescence (ECL, Millipore) on a ChemiDoc MP imaging system (Bio-Rad). Exposure times were optimized individually (30 s to 5 min) to prevent saturation. Band intensities were quantified with ImageJ, normalized to GAPDH, and expressed as fold-change relative to vector controls (set as 1.0). Experiments were independently repeated at least three times.

### Colony formation assay

Cells were trypsinized, counted, and seeded into 6-well plates at densities of 500 cells/well (OVCAR3) or 800 cells/well (A2780) in DMEM containing 2% fetal bovine serum (FBS). Medium was changed every three days. After 14 days, cells were fixed with 4% paraformaldehyde (PFA) for 20 min and stained with 0.1% crystal violet for 20 min at room temperature. Colonies containing ≥ 50 cells were manually counted under a microscope at 10× magnification. Experiments included three biological replicates (independent passages) with duplicate wells.

### Cell viability and IC_50_ determination

Cells were seeded in 96-well plates at 3 × 10^3^ cells/well. For proliferation assays, 10 μL of CCK-8 solution was added daily, and absorbance at 450 nm was measured after 1 h. For IC_50_ determination, cells were treated 24 h after seeding with serial dilutions of erastin (MedChemExpress, Cat# HY-15763). Erastin stock solution (10 mM) was prepared in DMSO, aliquoted, stored at −20 °C protected from light, and freshly diluted in culture medium (final DMSO concentration ≤ 0.1%). After 72 h incubation, 10 μL of CCK-8 solution was added, and absorbance at 450 nm was measured after 1 h. All experiments were repeated three times.

### Wound healing assay

Cells were seeded in 6-well plates and grown until reaching 100% confluence. To inhibit proliferation, cells were pretreated with mitomycin C (10 μg/mL, Targetmol) for 2 h. After washing with PBS, a scratch was created using a sterile 200-μL pipette tip. Cells were then rinsed again and cultured in medium containing 1% FBS. Images of the wound area were captured at 0 and 24 h using 4× magnification. Wound closure was quantified with ImageJ software and calculated as the percentage closure: [(initial area – final area)/initial area] × 100%. Three biological replicates, each performed in duplicate, were analyzed.

### Migration and invasion assays

Migration and invasion assays were conducted using 24-well Transwell chambers (8-μm pore size, Corning). For invasion assays, the upper chamber was pre-coated with Matrigel (50 μL; diluted 1:8 in serum-free medium) and incubated at 37 °C for 2 h. Cells (1 × 10^5^) in 200 μL of serum-free medium were seeded into the upper chamber, while the lower chamber contained 500 μL of medium with 10% FBS. After incubation for 24 h at 37 °C, non-invaded cells were gently removed using a cotton swab. Invaded cells were fixed with 4% paraformaldehyde for 20 min, stained with 0.1% crystal violet for 20 min, and imaged at 200× magnification (three random fields per insert). Migration assays were performed identically but without Matrigel coating. Three biological replicates, each performed in duplicate, were conducted for both assays.

### EdU proliferation assay

Cells were seeded onto 13-mm coverslips placed in 24-well plates (5 × 10^3^ cells/well) and incubated overnight to allow attachment. EdU stock solution (10 mM, UELandy kit) was diluted to 20 µM in culture medium and added to cells at a final concentration of 10 µM.

After incubation for 2 h at 37 °C, cells were fixed with 4% paraformaldehyde for 30 min, neutralized with 2 mg/mL glycine for 5 min, and permeabilized with 0.5% Triton X-100 for 10 min. EdU labeling was performed using the YF^®^ 594 Click-iT EdU Kit (UELandy, cat# C6048M) according to the manufacturer’s instructions. Nuclei were counterstained with Hoechst 33342 (5 µg/mL) for 30 min. Coverslips were mounted using anti-fade mounting medium.

Fluorescence images were captured at 200× magnification using an Olympus IX83 microscope (three random fields per coverslip). EdU-positive (red) and Hoechst-positive (blue) cells were counted using ImageJ software. EdU positivity rates were calculated as follows: (EdU-positive cells/total cells) × 100%. Experiments were repeated in three biological replicates, each with duplicate coverslips.

### Oil Red O staining

Cells were washed twice with PBS and fixed with Oil Red O fixative for 20–30 min. Following fixation, cells were rinsed twice with distilled water and then incubated with 60% isopropanol for approximately 30 s. Freshly prepared Oil Red O staining solution was added, and cells were stained for 10–20 min. After staining, cells were briefly rinsed again with 60% isopropanol until the background was clear, followed by several rinses with distilled water to remove excess dye. Nuclei were counterstained with Mayer’s hematoxylin for 1–2 min and rinsed with distilled water. Cells were then immersed in Oil Red O buffer for 1 min, rinsed once more with distilled water, and examined under a microscope. Imaged at 200× magnification (three random fields per insert).

### ROS detection

Cells were seeded into confocal dishes at a density of 1.5 × 10^5^ cells per dish and treated according to the experimental protocol. After treatment, medium was removed, and cells were washed gently three times with PBS (1 min per wash). Dihydroethidium (DHE; Beyotime, China) was diluted to 10 µM in serum-free medium, added to the cells (1 mL/dish), and incubated at 37 °C for 30 min in the dark (wrapped in aluminum foil). After incubation, cells were washed twice with PBS. Fluorescence images (three random fields per dish) were acquired using a Leica TCS SP8 confocal microscope (excitation, 488–535 nm; emission, 610 nm; red fluorescence indicating oxidized ethidium bound to DNA). Mean fluorescence intensity was quantified using ImageJ software and normalized to the control group (set as 1.0). Three independent biological replicates were performed.

### C11-BODIPY581/591 staining

Cells were seeded and incubated overnight at 37 °C. C11-BODIPY 581/591 was diluted from a 10 mM stock solution to a final concentration of 10 μM and added to the cells, followed by incubation at 37 °C for 30 min. After removing the medium, cells were washed three times with PBS. Hoechst 33342 solution was freshly prepared, protected from light, and added to cells for 30 min at room temperature on a shaker. Following staining, cells were washed with PBS (1–3 times). Fluorescence images (three random fields per dish) were acquired using a Leica TCS SP8 confocal microscope.

### Determination of Fe^2+^concentration

Intracellular Fe^2+^ concentrations were measured using an Iron Assay Kit (Colorimetric; Dojindo, cat# I291). Cells (2 × 10^5^ per well in 6-well plates) were treated according to experimental conditions, washed with PBS, scraped into 1.3 mL Assay Buffer, and transferred to microtubes. Samples were sonicated on ice for 5 min, centrifuged at 16,000 × g (10 min, 4 °C), and 400 µL supernatant was mixed with 20 µL Assay Buffer and incubated at 37 °C for 15 min. Subsequently, 200 µL of the mixture was transferred to a 96-well plate and incubated for an additional 60 min at 37 °C in the dark. Absorbance was measured at 593 nm. Fe^2+^ concentrations were calculated using a standard curve (0–100 µM Fe^2+^) and normalized to total protein content (nmol/mg). Three biological replicates, each with duplicate technical measurements, were performed.

### Malondialdehyde (MDA) assay

Intracellular MDA concentrations were determined using a lipid peroxidation (MDA) assay kit (Beyotime). Cells (2 × 10^5^ per well, 6-well plates) were treated, harvested, washed, and lysed in RIPA buffer. Protein concentrations were determined using a BCA assay. Briefly, samples, standards, or blanks (0.1 mL each) were mixed with MDA detection solution (0.2 mL) and heated in a boiling water bath for 15 min. After cooling and centrifugation (1000 × g, 10 min), 200 μL supernatant was transferred to a 96-well plate. Absorbance was read at 532 nm, and MDA concentrations (nmol/mg protein) were calculated from a standard curve. Three biological replicates, each performed in duplicate, were conducted.

### GSH measurement

Intracellular glutathione (GSH) levels were quantified using a GSH assay kit (Beyotime). Cells (2 × 10^5^ per well, 6-well plates) were treated, harvested, and washed with cold PBS, followed by resuspension in extraction buffer (200 μL/well). Samples were sonicated on ice (5 min) and centrifuged (8,000 × g, 10 min, 4 °C). Supernatants were collected, and 10 μL was mixed with 150 μL reaction mixture (containing DTNB and glutathione reductase) in a 96-well plate. After incubation at room temperature in the dark for 2 min, absorbance was measured at 412 nm. GSH concentrations were determined using a standard curve (0–20 μM) and normalized to protein content (nmol/mg). Three biological replicates with duplicate technical measurements were performed.

### RNA sequencing (RNA-seq)

Total RNA was extracted from OVCAR3 vector control and SLC44A4-overexpressing cells (*n* = 3 per group) using TRIzol reagent (Takara). RNA quality was verified by A260/A280 ratios (1.8–2.2) and agarose gel electrophoresis. Libraries were constructed by APExBIO (poly (A) enrichment, size selection 350–550 bp) and sequenced on an Illumina Xplus platform (PE150, approximately 30 M reads/sample). Raw reads were quality-filtered using Fastp and mapped to the GRCh38/hg38 genome using HISAT2. Gene counts were quantified with StringTie (GENCODE v38). Differential expression analysis was performed using DESeq2 with Benjamini–Hochberg correction; genes with |log_2_FC| > 1 and FDR < 0.05 were considered significantly different. GO/KEGG enrichment and Gene Set Enrichment Analysis (GSEA) were conducted using clusterProfiler (*p* < 0.05). Raw sequencing data are available in the OMIX016458 repository.

### Targeted lipidomics (free fatty acids)

Cells harvested during logarithmic growth phase were washed, trypsinized, pelleted (1000 × g, 1 min, 4 °C), snap-frozen, and stored at −80 °C. Samples (*n* = 3 per group) underwent derivatization with 1% H_2_SO_4_ in methanol (80 °C, 30 min), followed by extraction with n-hexane and addition of methyl salicylate as internal standard. Gas chromatography-mass spectrometry (GC-MS) analysis was performed on a Thermo Trace 1300-TSQ 9000 system with a TG-FAME column (50 *m* × 0.25 mm × 0.20 μm). The GC oven program was as follows: 80 °C for 1 min, ramped to 160 °C at 20 °C/min (1.5 min hold), then to 196 °C at 3 °C/min (8.5 min hold), and finally to 250 °C at 20 °C/min (3 min hold). Injection volume was 1 μL (split ratio 8:1), helium as carrier gas at 0.63 mL/min, operating in electron ionization-selected ion monitoring (EI-SIM) mode. Calibration curves with 51 fatty acid methyl ester (FAME) standards were established (R^2^ > 0.99; limit of quantification at signal-to-noise ratio = 10). Data were normalized to total protein content, log_2_-transformed, and median-normalized. Differential metabolites were identified by orthogonal partial least squares-discriminant analysis (OPLS-DA; VIP ≥ 1), t-test, and Benjamini-Hochberg correction (FDR < 0.1). Raw lipidomics data are accessible in the OMIX016459 repository.

### Animal experiments

Six-week-old female BALB/c nude mice (*n* = 5 per group; total *n* = 10) were purchased from Beijing Vital River Laboratory Animal Technology Co., Ltd. All procedures were approved by the Animal Research Center of The Affiliated Hospital of Qingdao University and conducted in accordance with the ARRIVE 2.0 guidelines [24,25]. Mice were randomly assigned to groups using a computer-generated random sequence, and allocation was concealed until treatment. Each mouse received a subcutaneous injection of 3 × 10^6^ OC cells in 100 μL of PBS. Tumor volumes were measured twice weekly from day 7 post-inoculation for 4 weeks by an investigator blinded to group allocation; group codes were revealed after data collection. Mice were anesthetized with isoflurane (induction at 3%, maintenance at 1.5% in O_2_) and euthanized by CO_2_ inhalation (20% chamber volume/min), followed by cervical dislocation.

### Expression level analysis

Gene and protein expression levels of SLC44A4 in OC and normal ovarian tissues were analyzed using the GEPIA (http://gepia.cancer-pku.cn) and Human Protein Atlas (https://www.proteinatlas.org) databases, respectively. Associations with tumor stage and p53 status were assessed using UALCAN (https://ualcan.path.uab.edu).

### Statistical analysis

All experiments were performed with at least three biological replicates (independent cell cultures). Technical replicates were averaged within each biological replicate prior to analysis. Normality was assessed using the Shapiro-Wilk test, and variance homogeneity using Levene’s test. For comparisons between two groups, a two-tailed Student’s t-test was applied for normally distributed data; otherwise, the Mann–Whitney U test was used. For comparisons involving three or more groups, one-way or two-way ANOVA was performed, followed by Tukey’s, Dunnett’s, or Šidák’s post hoc tests as appropriate. For non-normally distributed multi-group data, the Kruskal-Wallis test with Dunn’s post hoc correction was used. Data are presented as mean ± SD. Exact p-values were reported, and *p* < 0.05 was considered statistically significant. All analyses were conducted using GraphPad Prism 9.0.

## Results

### SLC44A4 is highly expressed in OC and is associated with disease progression and poor prognosis

**Figure 1.**
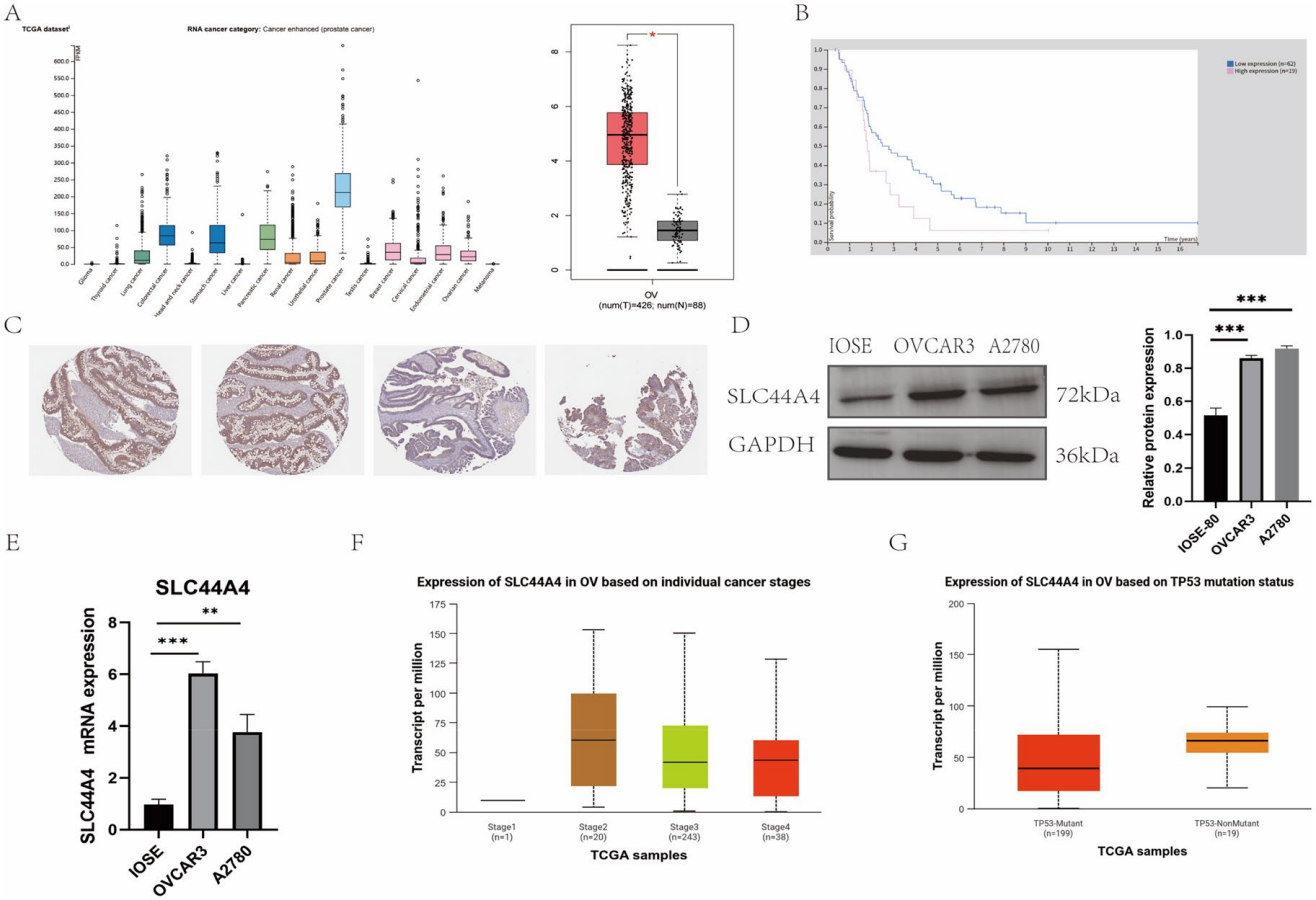
(A) Pan-cancer expression profile of SLC44A4 (HPA) and its expression in OC (GEPIA). (B) Kaplan–Meier survival curve showing the association between SLC44A4 expression and overall survival in OC patients (HPA). (C) Representative IHC images of SLC44A4 protein expression in OC tissues (HPA). (D) Western blot analysis of SLC44A4 protein levels in IOSE80, OVCAR3, and A2780 cells. (E) qRT-PCR analysis of SLC44A4 mRNA expression in the same cell lines. (F) SLC44A4 expression stratified by tumor stage (UALCAN). (G) SLC44A4 expression stratified by p53 mutation status (UALCAN). (*p < 0.05, **p < 0.01, ***p < 0.001.).

According to the Human Protein Atlas (HPA), SLC44A4 exhibits variable expression across cancer types, with high expression levels observed in gastrointestinal and gynecological malignancies (Figure 1A). Immunohistochemistry (IHC) data from the HPA confirmed SLC44A4 protein expression in OC tissues (Figure 1C). Analysis of the GEPIA database showed that SLC44A4 expression was significantly higher in OC tissues than in normal ovarian tissues (Figure 1A). Moreover, patients with elevated SLC44A4 expression exhibited poorer overall survival compared with those with lower expression (Figure 1B). At the cellular level, Western blotting and qRT-PCR analyses demonstrated that both protein and mRNA levels of SLC44A4 were upregulated in OVCAR3 and A2780 cells relative to IOSE80 cells (Figure 1D, E). Analysis using the UALCAN database further indicated that SLC44A4 expression varied across tumor stages (Figure 1F) and was also associated with p53 mutation status (Figure 1G).

### SLC44A4 overexpression promotes OC cell proliferation and invasion in vitro and in vivo

**Figure 2.**
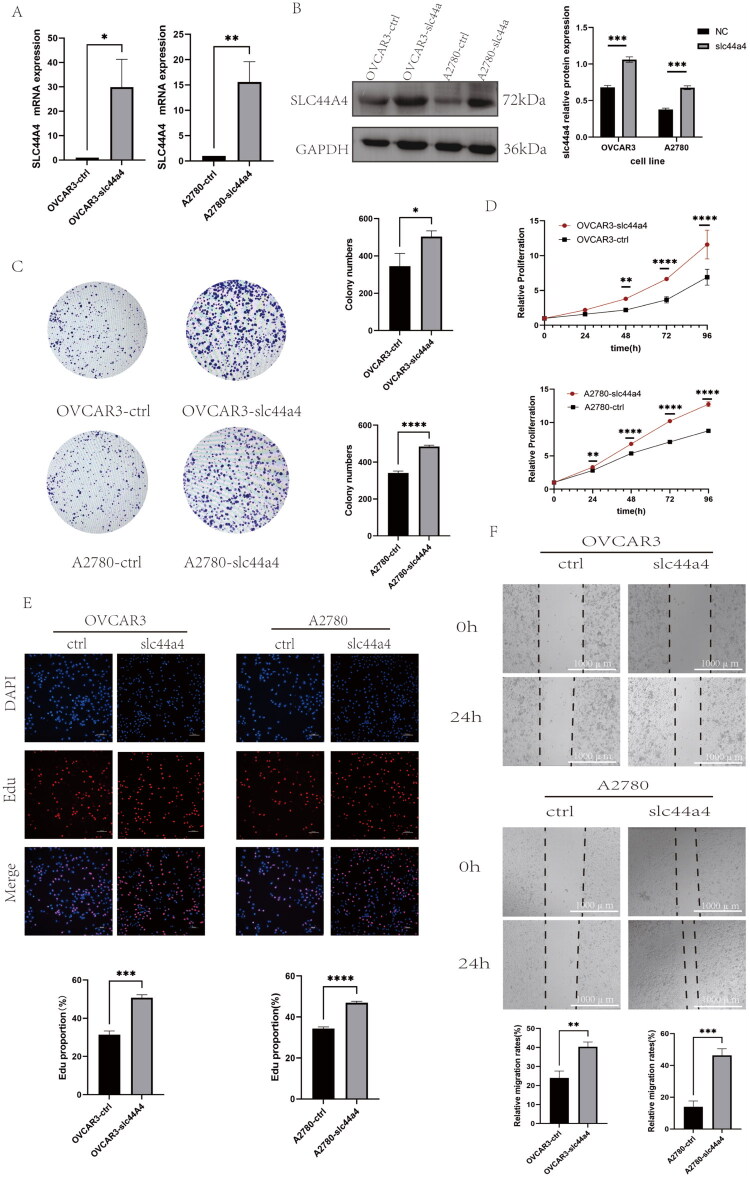
(A) RT-qPCR validation of SLC44A4 mRNA expression in OVCAR3 and A2780 cells following lentiviral transduction. (B) Western blot validation of SLC44A4 protein expression in the same cells. (C) Colony formation assay showing increased colony-forming capacity after SLC44A4 overexpression. (D) CCK-8 assay demonstrating enhanced proliferation. (E) EdU assay confirming increased DNA synthesis. Scale bar, 100 μm. (F) Wound-healing assay evaluating the migratory capacity of EOC cells. Scale bar, 1000 μm (*p < 0.05, **p < 0.01, ***p < 0.001, ****p < 0.0001).

**Figure 3.**
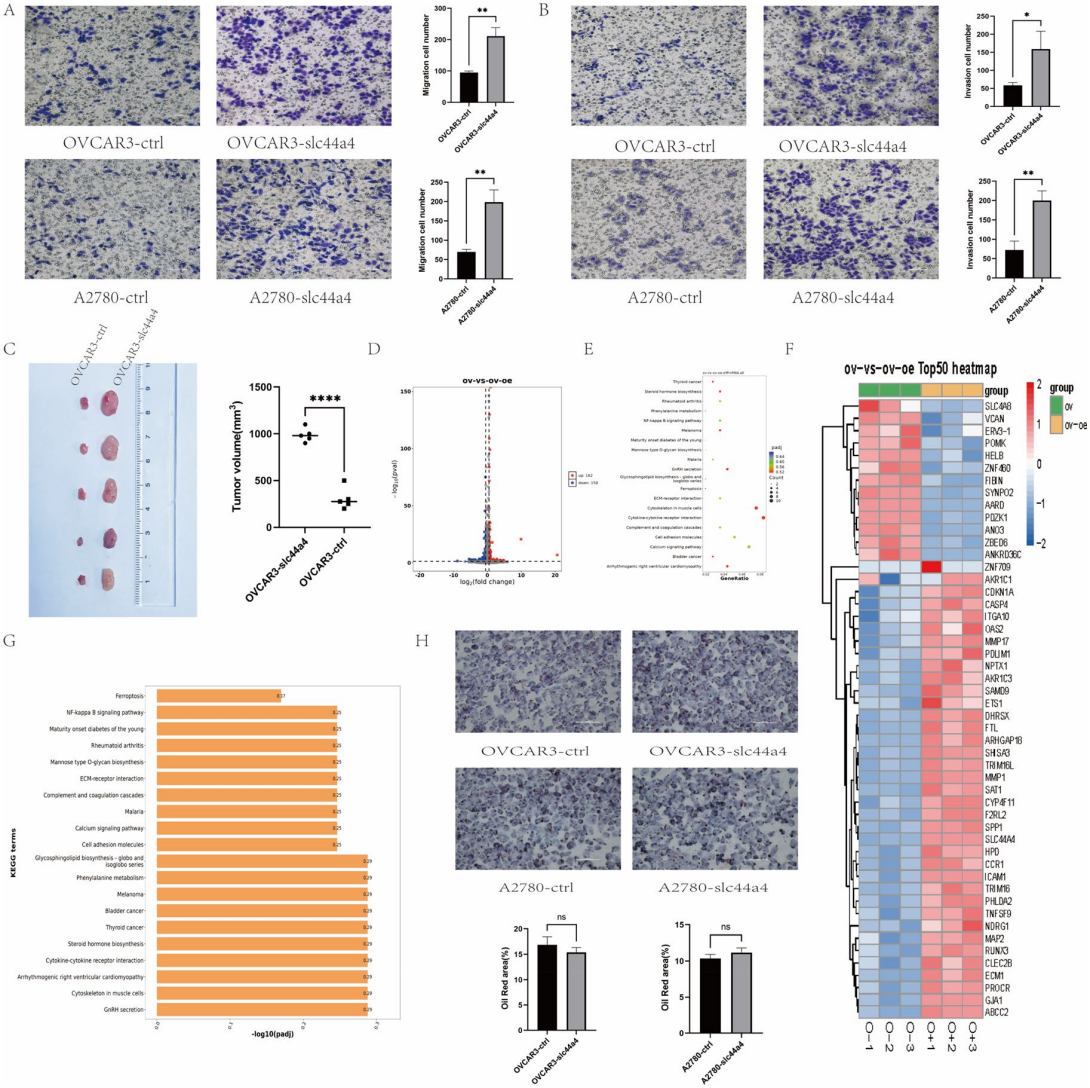
(A) Migration assay (without Matrigel) to assess cell migration. Scale bar, 100 μm. (B) Invasion assay (with Matrigel) to assess cell invasion. Scale bar, 100 μm. (C) Representative images of subcutaneous tumors formed by OVCAR3 cells with or without SLC44A4 overexpression, with corresponding quantitative analysis of tumor volume. (D) Volcano plot showing differentially expressed genes following SLC44A4 overexpression (|log_2_FC| > 1, FDR < 0.05). (E) Scatter plot of KEGG pathway enrichment analysis. (F) Heatmap of differentially expressed genes. (G) Bar plot of enriched KEGG pathways. (H) Oil Red O staining of lipid droplets; no significant difference was observed following SLC44A4 overexpression. Scale bar, 100 μm (*p < 0.05, **p < 0.01, ****p < 0.0001).

Stable OVCAR3 and A2780 cell lines with SLC44A4 overexpression were established to investigate its role in OC, and overexpression efficiency was confirmed (Figure 2A,B). Functional assays, including CCK-8 (Figure 2D), EdU (Figure 2E), and colony formation (Figure 2C), showed that SLC44A4 overexpression significantly enhanced cell proliferation and colony-forming ability in both cell lines compared with controls. In addition, wound-healing (Figure 2F) and Transwell assays (Figure 3A,B) demonstrated increased migration and invasion following SLC44A4 overexpression. In a subcutaneous xenograft model, SLC44A4 overexpression significantly promoted tumor growth *in vivo* (Figure 3C). These findings indicate that SLC44A4 overexpression enhances OC cell proliferation and invasion both *in vitro* and *in vivo*.

### SLC44A4 overexpression reshapes the cellular fatty acid profile without influencing lipid droplet formation

To investigate the mechanisms underlying SLC44A4-mediated regulation of OC cell proliferation and migration, RNA sequencing (RNA-seq) was performed. A total of 312 genes were differentially expressed following SLC44A4 overexpression, including 162 upregulated and 150 downregulated genes (Figure 3D). The top 50 enriched genes are presented in Figure 3F. Among these, several genes related to cell adhesion, including VCAN [26], POMK [27], ITGA10 [28], and PDLIM1 [29], were identified. Tumor suppressor genes such as SHISA3 [30] and TRIM16L [31] were upregulated. Additionally, genes associated with lipid metabolism or ferroptosis, including CYP4F11 [32], PHLDA2 [33], CLEC2B [34], ABCC2 [35], and FTL [36], showed altered expression (Figure 3E,G).

**Figure 4.**
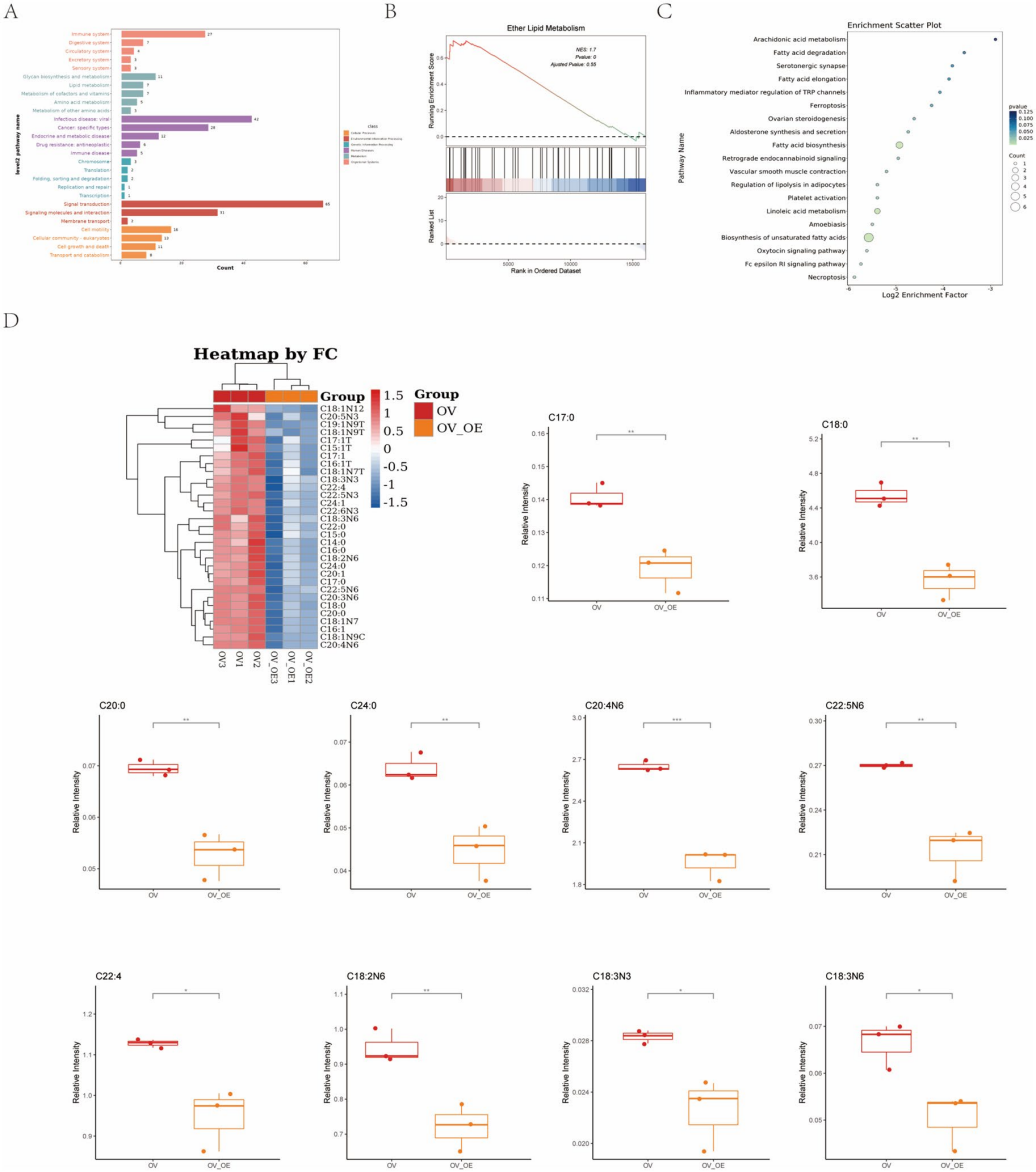
(A) Bar plot showing the top 5 enriched KEGG pathways. (B) GSEA of GO terms following SLC44A4 overexpression. (C) Scatter plot of KEGG pathway enrichment for metabolic pathways. (D) Heatmap and bar plot of differentially expressed metabolites (*p < 0.05, **p < 0.01, ***p < 0.001).

Given previous reports linking SLC44A4 to blood lipid levels [37], we further explored its role in lipid metabolism. Transcriptome analysis supported an association between SLC44A4 and lipid-related pathways (Figure 4A,B). Accordingly, targeted lipidomics was performed, revealing significantly reduced levels of saturated fatty acids (C17:0, C18:0, C20:0, C24:0) and polyunsaturated fatty acids in SLC44A4-overexpressing cells compared with controls (Figure 4D). In contrast, Oil Red O staining demonstrated no significant differences in lipid droplet content between these groups (Figure 3H). Collectively, these data indicate that SLC44A4 overexpression alters cellular fatty acid composition without affecting lipid droplet synthesis.

### Ferroptosis and lipid peroxidation are attenuated by SLC44A4 overexpression in OC cells

**Figure 5.**
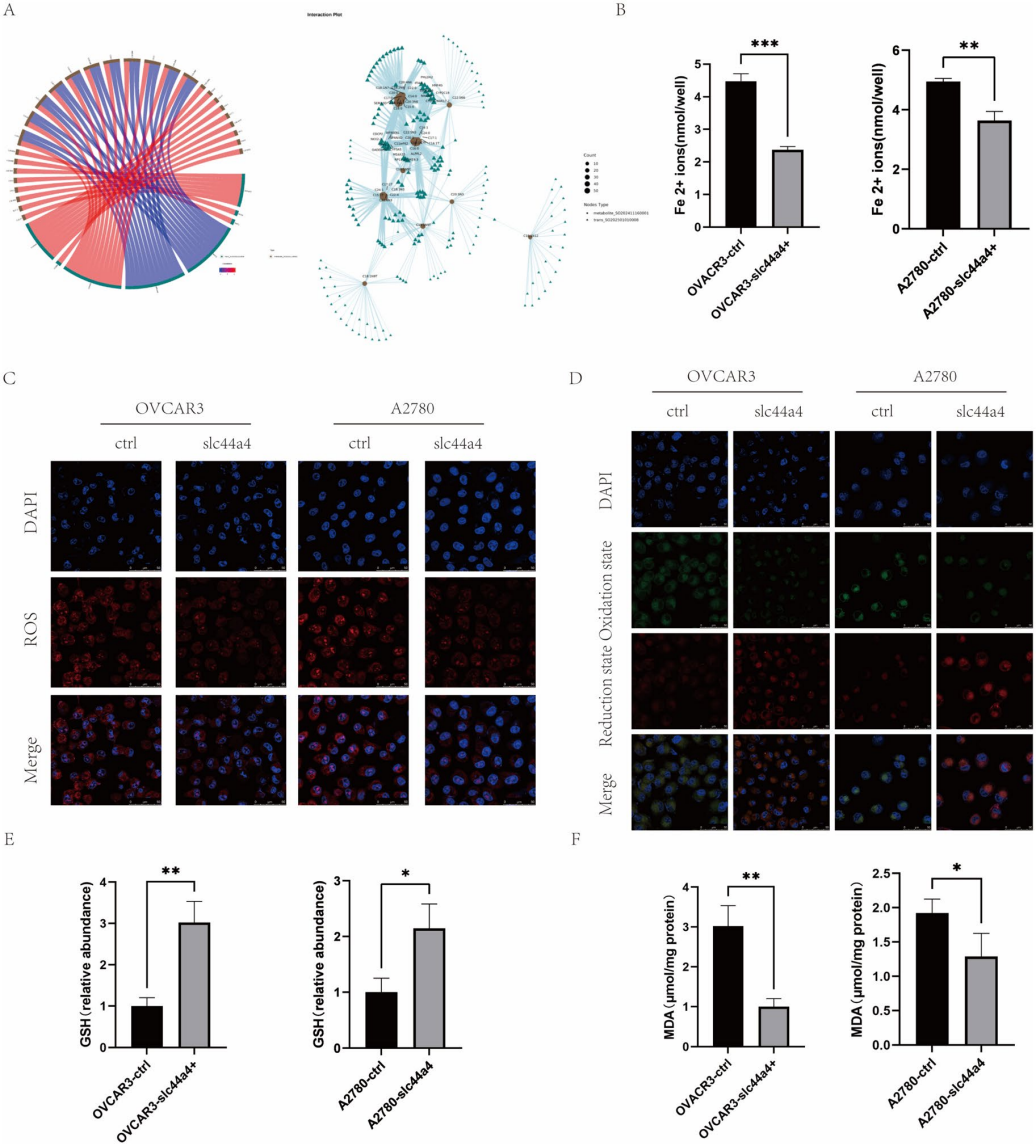
(A) Correlation chord diagram and network diagram integrating transcriptomic and lipidomic data. (B) Measurement of Fe^2+^ levels in OVCAR3‑ctrl, OVCAR3‑SLC44A4, A2780‑ctrl, and A2780‑SLC44A4 cells. (C) Confocal microscopy of intracellular ROS levels in the same groups. Scale bar, 50 μm. (D) Confocal microscopy of intracellular lipid peroxidation (C11‑BODIPY581/591). Scale bar, 50 μm. (E) GSH levels in the indicated groups. (F) MDA levels in the same groups (*p < 0.05, **p < 0.01, ***p < 0.001).

KEGG pathway enrichment analysis suggested involvement of ferroptosis-related pathways in lipid metabolism (Figure 4C). Integrated RNA-seq and targeted lipidomics analysis further identified differential expression of SIDT1 and CYP3A5 between OVCAR3-control and OVCAR3-SLC44A4-overexpressing cells (Figure 5A). SIDT1 is a transmembrane phospholipase involved in nucleic acid uptake [38]. CYP3A5, a cytochrome P450 family member, catalyzes drug metabolism and synthesizes lipids such as cholesterol and steroids, producing reactive oxygen species (ROS) during catalysis [39,40].

To determine whether SLC44A4 influences ferroptosis, we assessed cellular lipid ROS levels using a ROS detection kit (Figure 5C) and lipid peroxidation using a C11‑BODIPY581/591 assay (Figure 5D). SLC44A4 overexpression significantly reduced lipid ROS levels and lipid peroxidation. Because Fe^2+^ accumulation is an early marker of ferroptosis [41], intracellular iron levels were measured. Fe^2+^ accumulation was significantly reduced in SLC44A4‑overexpressing cells compared with controls (Figure 5B). MDA, a final product of lipid peroxidation, and GSH, a key antioxidant, were also measured [42]. SLC44A4 overexpression decreased MDA levels and increased GSH levels (Figure 5E,F), consistent with a protective effect against ferroptosis.

**Figure 6.**
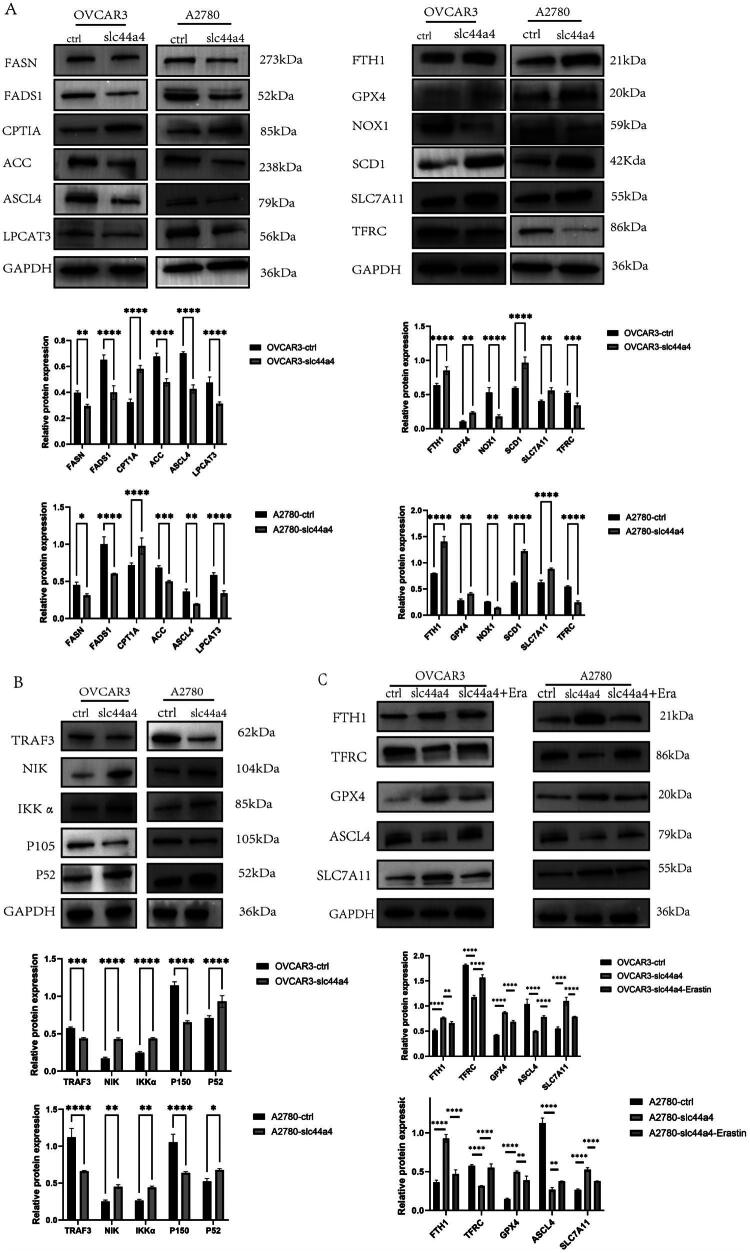
(A) Western blot analysis of ferroptosis‑related proteins in OVCAR3 and A2780 cells following SLC44A4 overexpression. (B) Western blot analysis of non‑canonical NF‑κB pathway components in the same cells. (C) Western blot analysis of ferroptosis‑related proteins in SLC44A4‑overexpressing cells after erastin treatment. (*p < 0.05, **p < 0.01, ***p < 0.001, ****p < 0.0001.).

Next, the expression of ferroptosis‑related proteins was examined. Western blot analysis showed that, compared with OVCAR3‑ctrl and A2780‑ctrl cells, OVCAR3‑SLC44A4 and A2780‑SLC44A4 cells exhibited increased levels of ferroptosis‑inhibitory proteins and decreased levels of ferroptosis‑promoting proteins (Figure 6A). As shown in Figure S1, treatment with Fer‑1 (1  μM) significantly reversed erastin‑induced loss of cell viability in SLC44A4‑overexpressing cells. Overall, SLC44A4 overexpression attenuates ferroptosis in OC cells and is associated with reduced lipid peroxidation.

### Overexpression of SLC44A4 suppresses ferroptosis through non-canonical NF-κB signaling in a NIK-dependent manner

**Figure 7.**
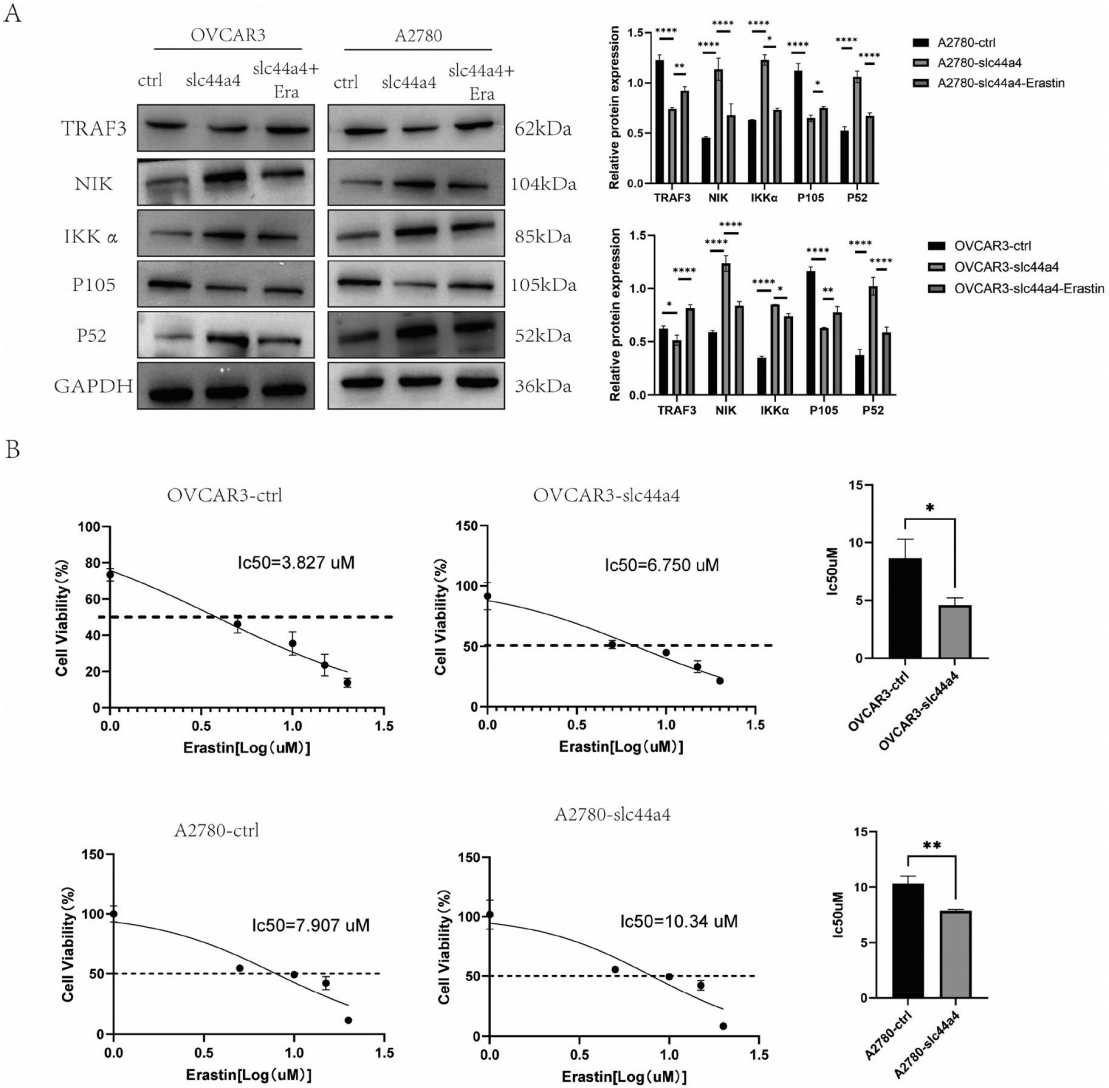
(A) Western blot analysis of non‑canonical NF‑κB pathway components in OVCAR3 and A2780 cells after erastin treatment with or without SLC44A4 overexpression. (B) IC_50_ values of erastin in OVCAR3 and A2780 cells with or without SLC44A4 overexpression (*p < 0.05, **p < 0.01, ****p < 0.0001).

RNA‑seq data indicated that SLC44A4 regulates the NF‑κB pathway (Figure 3G). Consistently, Western blot analysis showed that SLC44A4 overexpression reduced TRAF3 and p105 levels while increasing IKKα, NIK, and p52 expression (Figure 6B). Following treatment with the ferroptosis inducer erastin in SLC44A4‑overexpressing OVCAR3 and A2780 cells, both ferroptosis‑related proteins and components of non‑canonical NF‑κB signaling were altered (Figures 6C and 7A). To further assess pathway dependency, SLC44A4‑overexpressing cells were pretreated with the NIK inhibitor B022 (5 μM) before erastin exposure. As shown in Fig. S2, B022 (5 μM) eliminated the protective effect of SLC44A4 overexpression. These results suggest that SLC44A4 overexpression suppresses ferroptosis *via* non‑canonical NF‑κB signaling.

### Overexpression of SLC44A4 reduces the sensitivity of OC cells to the ferroptosis inducer erastin

Erastin is a well‑established inducer of ferroptosis. To evaluate whether SLC44A4 affects ferroptosis sensitivity, OC cells were treated with increasing concentrations of erastin (0, 1, 10, 15, and 20 µM) for 72 h. Cell viability was then measured using a CCK‑8 assay, and the half‑maximal inhibitory concentration (IC_50_) was calculated. SLC44A4 overexpression resulted in a higher IC_50_ compared with controls (Figure 7B), indicating reduced sensitivity to erastin‑induced ferroptosis.

## Discussion

The solute carrier (SLC) family is the second-largest family of membrane proteins, following G-protein-coupled receptors. SLC proteins transport diverse substrates, including small molecules, across biological membranes and have attracted increasing attention recently. SLC44A4 is believed to participate in acetylcholine synthesis and transport, as well as the uptake of thiamine pyrophosphate (the active form of vitamin B1) [15]. Our transcriptomic data indicate that besides ferroptosis, SLC44A4 may influence apoptosis and cell adhesion. We identified altered expression of cell adhesion-related factors, such as VCAN [43], ITGA10 [28], and PDLIM1 [29], as well as CASP4, a key regulator of autophagy‑pyroptosis [44]. SLC44A4 is highly expressed in OC cells and promotes their proliferation, with expression closely linked to altered tumor suppressors such as SHISA3 [45], TRIM16 [46], and CDKN1A [47]. Recently, SLC44A4 has been explored as a target for antibody-drug conjugates in prostate and pancreatic cancers, with promising clinical responses in early trials [17,48]. Although further clinical applications have not yet advanced, these studies highlight the therapeutic potential of SLC44A4.

OC is among the most heterogeneous malignancies, with rapidly increasing global incidence. Despite advances in multidisciplinary treatment, overall patient survival remains poor [49]. Growing evidence indicates that OC cells develop resistance to multiple therapies by reprogramming metabolic pathways or altering epigenetic signatures, ultimately leading to treatment failure [50]. Recent studies suggest OC, characterized by enhanced lipid metabolism, is particularly sensitive to ferroptosis [9,51]. Thus, ferroptosis-targeted therapies may represent a promising approach for OC treatment.

Although targeting metabolic reprogramming has emerged as a promising cancer therapy, the mechanisms through which SLC44A4 regulates ferroptosis remain unclear. Our RNA-seq analysis revealed that SLC44A4 overexpression reduces transcript levels of TRAF3, an upstream regulator of non-canonical NF-κB signaling. TRAF3 activates p105 and p52, thereby influencing cell proliferation and lipid metabolism [52,53]. Our study identified SLC44A4 as a novel regulator of ferroptosis in OC cells. By negatively regulating TRAF3, SLC44A4 alters cellular lipid metabolism, reduces polyunsaturated fatty acid abundance, and suppresses ferroptosis.

However, our findings are based solely on SLC44A4 overexpression models. Loss-of-function studies, validation of TCGA associations in independent cohorts, testing additional ferroptosis inducers (e.g. RSL3), and further exploration of non-canonical NF-κB signaling (nuclear p52/RelB translocation, TRAF3 rescue, IKKα silencing) are required. Additionally, the limited sample size restricts the generalizability of these results, necessitating cautious interpretation. These limitations will be addressed in future research. Furthermore, we speculate that SLC44A4 may influence ferroptosis through p53, as RNA-seq revealed changes in CDKN1A, a well-established p53 target [54].

In summary, our study demonstrates that SLC44A4 overexpression reduces lipid peroxidation and inhibits ferroptosis in OC cells, an effect associated with activation of non-canonical NF-κB signaling. SLC44A4 may therefore serve as a promising candidate for further investigation into ferroptosis modulation in OC, although its therapeutic potential requires additional validation.

## Supplementary Material

Supplementary_figure.docx

## Data Availability

Data will be made available upon reasonable request.
