# Supplementary material for: Overexpression of SLC44A4 suppresses ferroptosis and reduces lipid peroxidation via noncanonical NF-κB signaling in a NIK-dependent manner
Source: Ann Med. 2026 May 25;58(1):2677931. doi: 10.1080/07853890.2026.2677931 (PMC13215434; doi:10.1080/07853890.2026.2677931)
Supplement: Supplementary_figure.docx [file IANN_A_2677931_SM9653.docx]

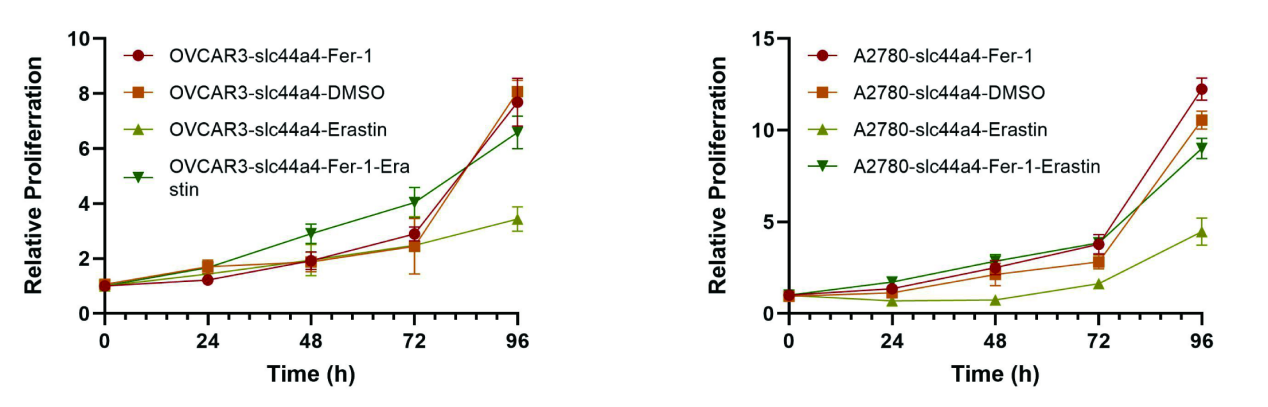


SFigure 1 Verification of ferroptosis specificity by rescue with Ferrostatin‑1 (Fer‑1) in SLC44A4‑overexpressing ovarian cancer cells using CCK‑8 assay.


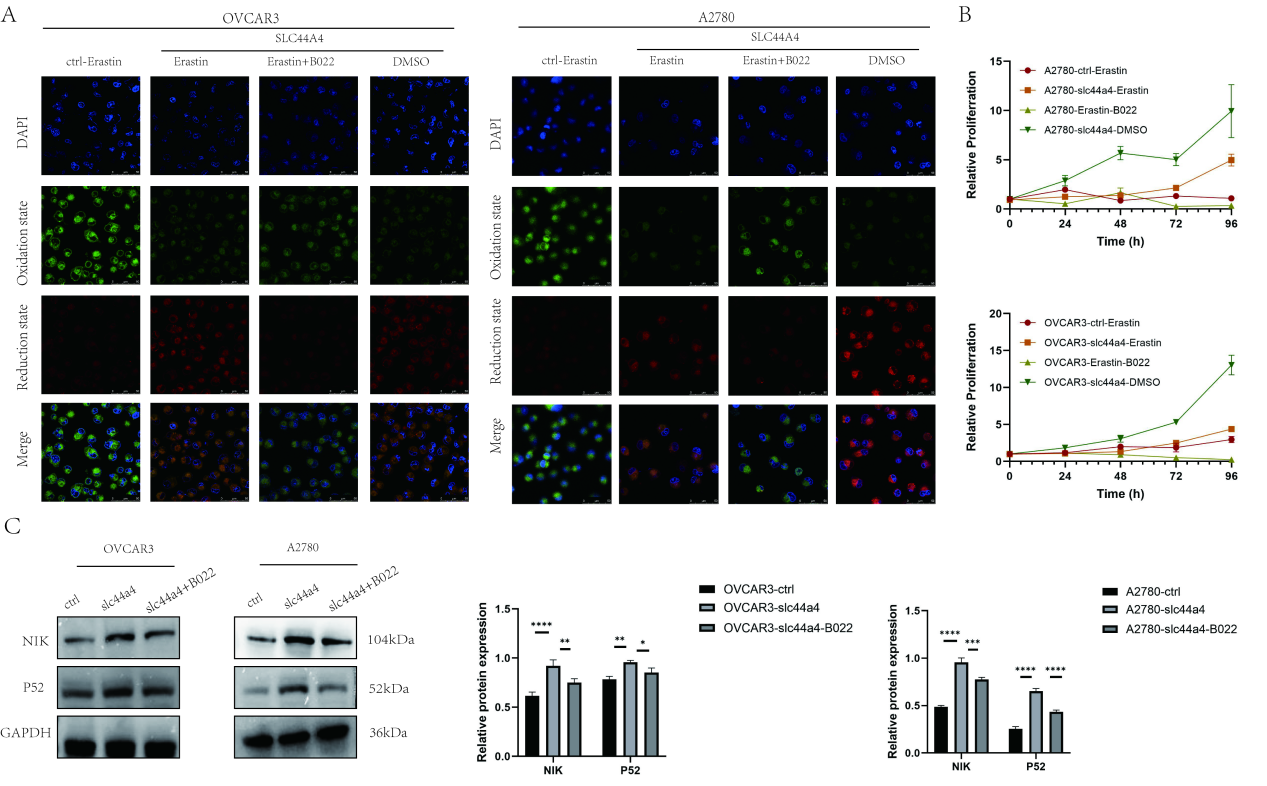


SFigure 2 (A) Lipid peroxidation (C11‑BODIPY) is increased by B022. (B) CCK‑8 viability assay showing that B022 (5 μM) eliminates the protective effect of SLC44A4 overexpression. (C) Western blots demonstrating that B022 inhibits NIK and downstream p52.
